# Developmental Table and Three-Dimensional Embryological Image Resource of the Ascidian *Ascidiella aspersa*

**DOI:** 10.3389/fcell.2021.789046

**Published:** 2021-12-17

**Authors:** Haruka M. Funakoshi, Takumi T. Shito, Kotaro Oka, Kohji Hotta

**Affiliations:** ^1^ Department of Biosciences and Informatics, Faculty of Science and Technology, Keio University, Yokohama, Japan; ^2^ Waseda Research Institute for Science and Engineering, Waseda University, Shinjuku, Japan; ^3^ Graduate Institute of Medicine, College of Medicine, Kaohsiung Medical University, Kaohsiung City, Taiwan

**Keywords:** *Ascidiella aspersa*, CLSM, developmental stage, 3D anatomy, transparent, tail bending, bioimaging

## Abstract

*Ascidiella aspersa* is an ascidian in the class of chordates—the closest relatives of vertebrates. *A*. *aspersa* is a potential model organism for bio-imaging studies due to its extremely transparent embryos as well as is a globally distributed cosmopolitan species. However, there is no standard developmental table for this organism. Here, as a first step to establish *A*. *aspersa* as a model organism, we report a standard developmental table as a web-based digital image resource. This resource used confocal laser scanning microscopy to scan more than 3,000 cross-sectional images and 3D-reconstructed images of *A*. *aspersa* embryos during embryogenesis. With reference to the standardized developmental table of *Ciona intestinalis* type A, 26 different developmental stages (Stages 1–26) from fertilized eggs to hatched larvae were redefined for *A*. *aspersa*. Cell lineages up to the cleavage period were annotated: The cleavage patterns, the embryonic morphology, and the developmental time were then compared with *Ciona*. We found that the cleavage patterns and developmental time up to the neurula period in *A*. *aspersa* were extremely conserved versus. *Ciona*. The ratio of the trunk and tail length in the tailbud period were smaller than *Ciona* indicating a relatively short tail. In addition, the timing of the bending of the tail is earlier than *Ciona*. This *A*. *aspersa* standard 3D digital resource is essential for connecting different omics data to different spatiotemporal hierarchies and is useful for a system-level understanding of chordate development and evolution.

## Introduction

Solitary ascidians such as *Halocynthia roretzi*, *Ciona intestinalis*, and *Phallusia mammillata* develop mosaics and are good model organisms to understand chordate developmental mechanisms. These ascidians belong to different genera but show stereotypical and similar cleavage patterns (Nishida, 1987; Lemaire, 2009; McDougall et al., 2011).

*Ascidiella aspersa* is an invasive tunicate found worldwide and devastating biofouler to shellfish aquaculture operations (Lynch et al., 2016; Palanisamy et al., 2018). Phylogenetic analysis by mitochondrial and COI mixed sequence analysis has suggested that *Ascidiella aspersa* belongs to family Ascidiidae including *Ascidia zara*, *Phallusia mammillata*, *Phallusia fumigata*, and *Ascidiella scabra*. *Ascidiella scabra* is closest to *Ascidiella aspersa* (Shito et al., 2020). The high biotic potential of *Ascidiella aspersa* bioinvaders can have a negative impact on native fauna in an introduced ecosystem due to its highly efficient reproductive and resource allocation strategies (Lynch et al., 2016). *A*. *aspersa* inhabits Japanese coasts. *A*. *aspersa* was first found as an alien species in 2008 from Funka Bay, Hokkaido, northern Japan leading to serious damage to the scallop aquaculture industry (Kanamori, 2016). Thus, *A*. *aspersa* is distributed globally.

Recent hyper-spectral imaging analysis by Shito et al. (2020) revealed that *A*. *aspersa* has one of most transparent eggs among solitary ascidians. The Ascidiidae family may have selective pressure for higher egg transparency. *Ascidiella aspersa* showed extremely high (88.0 ± 1.6%) bio-transparency in eggs that were maintained in the “invisible” larva. The embryological transparency is advantageous for bio-imaging studies as seen in transparent embryos of *Phallusia mammillata* (Robin et al., 2011b; Yasuo and McDougall, 2018), which is distributed mainly in Europe. The very transparent embryos of *A*. *aspersa* are a huge advantage for researchers who are not near the Mediterranean Sea and do not have ready access to *Phallusia*. Therefore, *A*. *aspersa* shows the potential to be an excellent model organism for bioimaging analysis.

Early embryonic staging of the ascidian *Halocynthia roretzi*, *Ciona intestinalis*, and *Phallusia mammillata* have been well described (Nishida, 1987; Hotta et al., 2007; Leggio et al., 2019; Dardaillon et al., 2020; Hotta et al., 2020). While the definition of the developmental stage is required to be applicable across species, the fact that there are different parts within the same developmental stage makes us recognize the difference as a species: Identical stages of different species’ embryos are often identified by different body lengths or shapes. After determining the precise annotation of developmental stage based on detailed images, these stages can then be compared to the species’ specific morphology. This requires temporally diverse images that contain information on the morphology of the embryo’s interior cells and the exterior surface cells.

The purpose of this study is to construct a standard 3D developmental table and image resource of embryogenesis by annotating the morphology at each defined stage of the *A*. *aspersa* embryo. Thus, we collected CLSM images of developing *A*. *aspersa* embryos sequentially at a stable temperature (20°C). This was combined with a time-lapse movie (Supplementary Movie S1). Morphometrical information about the length of the tail, trunk ratio, and aspect ratio of notochord cells are described for each stage.

We also constructed the Resources of *Ascidiella aspersa* Morphology Network-based as R*A*MNe (https://www.bpni.bio.keio.ac.jp/RAMNe/latest/index.html). This approach offers 3D anatomical information at the cellular level in various embryonic stages of *A*. *aspersa* to facilitate a system level understanding of morphology and evolution in invertebrate chordates ascidians (SLUMEICA).

## Results

### Developmental Staging of *Ascidiella aspersa*

**FIGURE 1:**
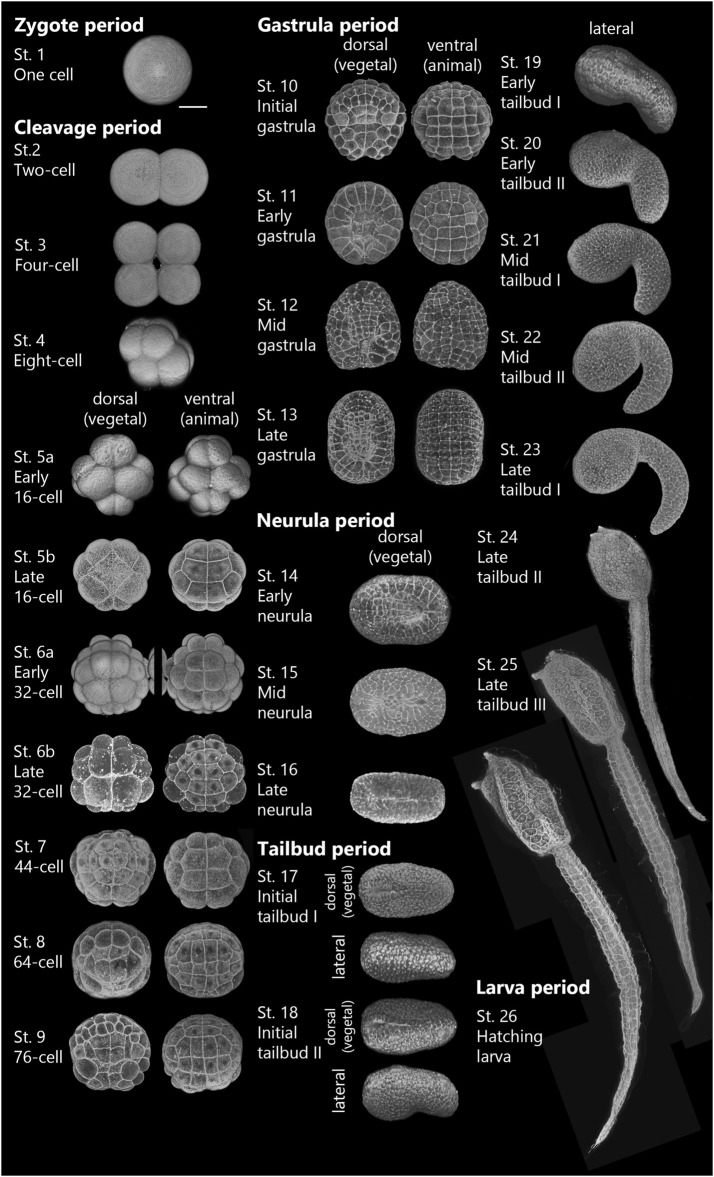
Three-dimensional reconstructed images of the *Ascidiella aspersa* embryo in the developmental time course after fertilization. Fertilized eggs were dechorionated and incubated at 20°C. Embryos were stained with Alexa Fluor™ 546 Phalloidin. In Stages 1–13, the anterior side of each embryo is up, and the anterior side is on the left in Stages 14–26. Stage 1 is in the zygote period. Stages 2–9 are in the cleavage period. Stages 10–13 are in the gastrula period. Stages 14–16 are in neurula period. Stages 17–25 are in the tailbud period. Stage 26 is in larva period. See the criteria for each stage in Table 1. Scale bar: 50 μm.

**FIGURE 2:**
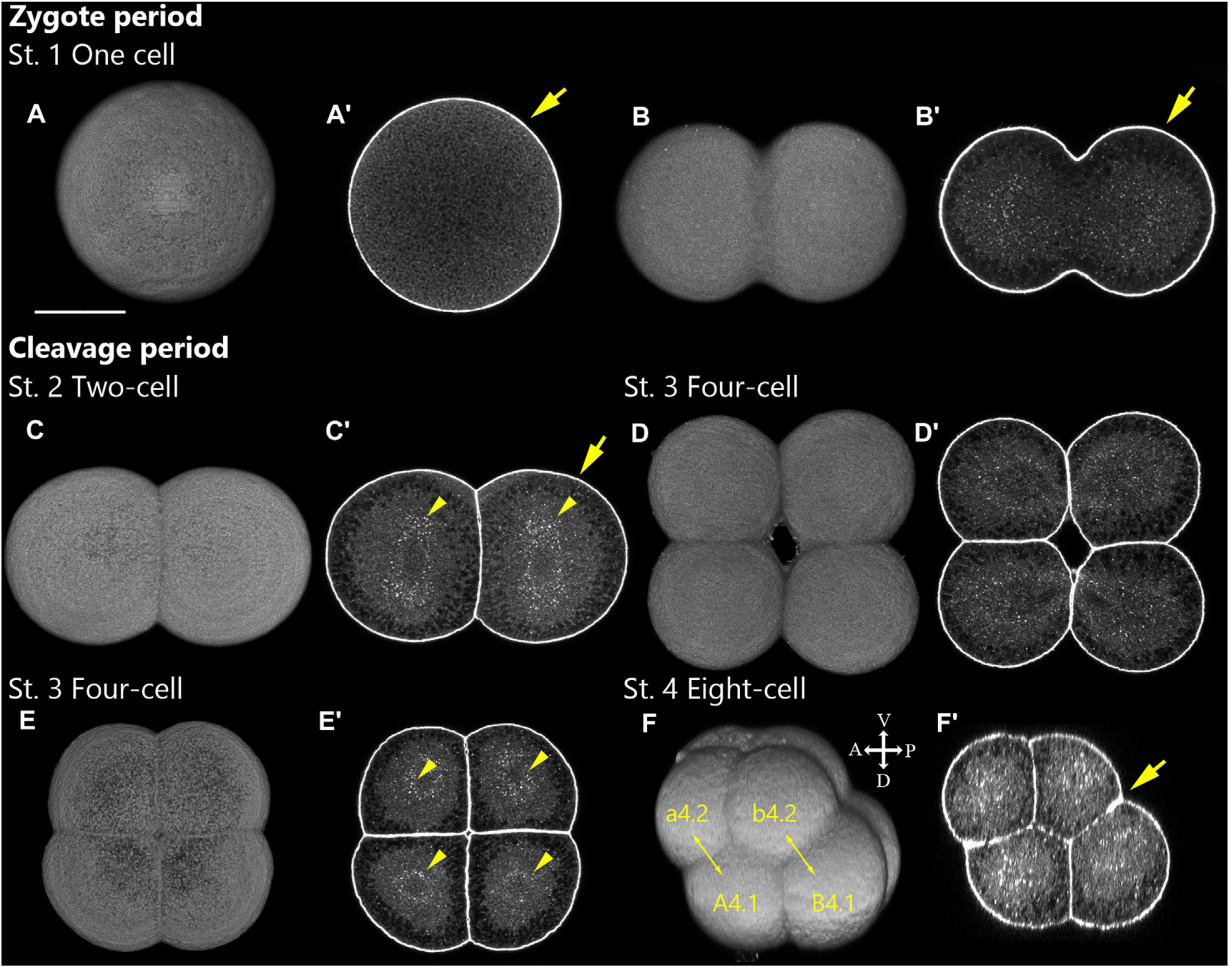
*Ascidiella aspersa* embryos at zygote and the initial part of cleavage periods. Embryos are stained with Alexa Fluor™ 546 Phalloidin. Three-dimensional reconstructed images at **(A,B)** Stage 1 (One cell), **(C)** Stage 2 (2-cell), **(D,E)** Stage 3 (4-cell), and **(F)** Stage 4 (8-cell). **(A′–F′)** The cross-section of **(A–F)**, respectively. Anterior side of each embryo is up unless noted specially in the body axis. In **(F,F′)**, embryo direction is shown by A, anterior; P, posterior; D, dorsal; V, ventral. In addition to the cortical actin filaments beneath the cell membranes (arrows), the staining could detect the cytosolic actin filaments surrounding the nuclei (arrowheads). Two-headed arrows indicate a pair of daughter cells. The number of cell-lineages are drawn on each blastomere. Scale bar: 50 μm.

**FIGURE 3:**
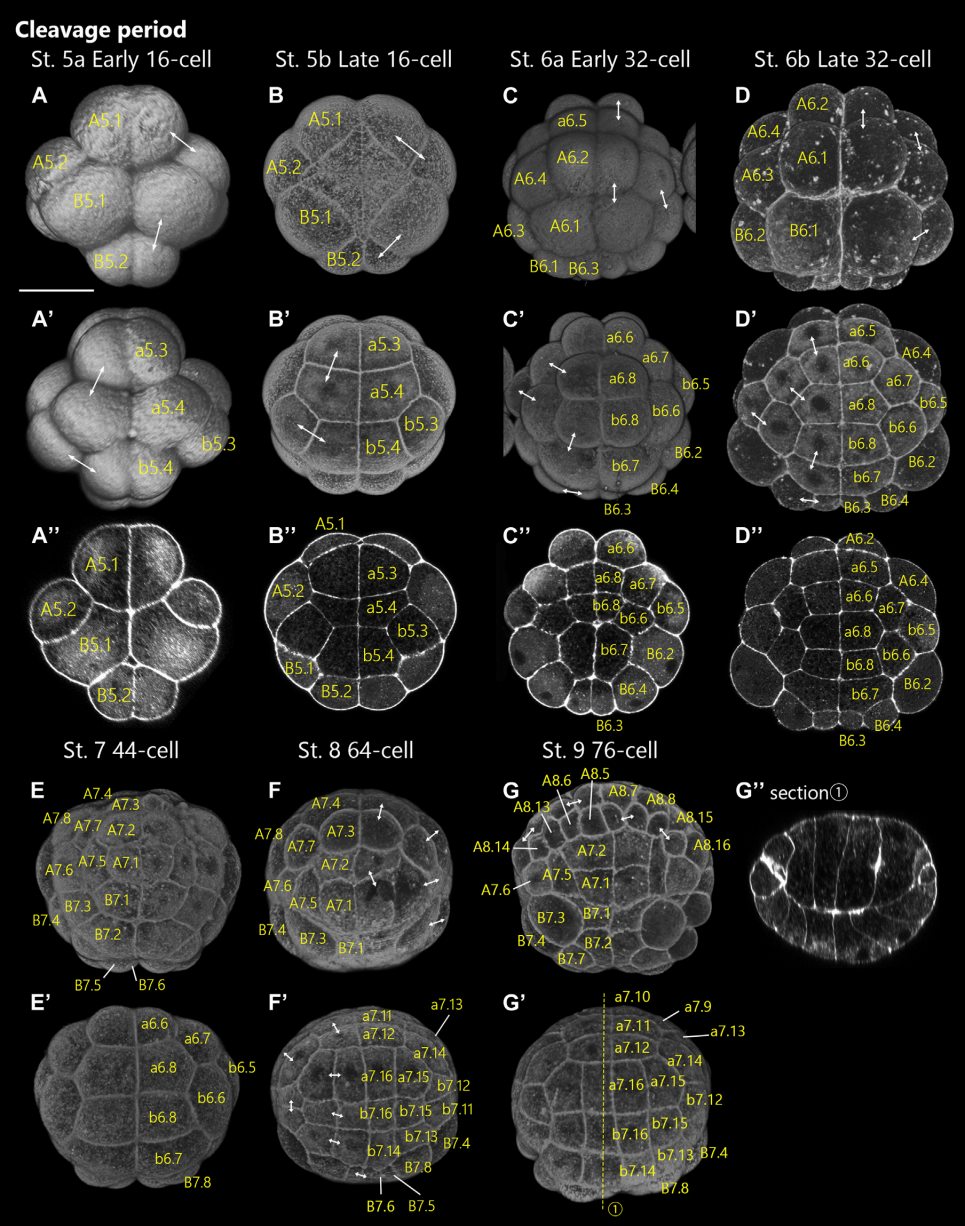
*Ascidiella aspersa* embryos at the latter of cleavage period. Embryos are stained with Alexa Fluor™ 546 Phalloidin. Three-dimensional reconstructed images at **(A,A′)** Stage 5a (early 16-cell), **(B,B′)** Stage 5b (late 16-cell), **(C,C′)** Stage 6a (early 32-cell), **(D,D′)** Stage 6b (late 32-cell), **(E,E′)** Stage 7 (44-cell), **(F,F′)** Stage 8 (64-cell), and **(G,G′)** Stage 9 (76-cell). **(A–G)** show the dorsal views of the embryos, and **(A′–G′)** show the ventral views of the embryos. Panels **(A′′-D′′)** are cross-sections of **(A–D)**, respectively. **(G′′)** shows longitudinal sections of **(G,G′)**. The positions of the sections are indicated by dashed lines in **(G′)**. Anterior side of each embryo is up except for **(G′′)** (vegetal side is up in **(G′′)**. Two-headed arrows indicate the pair of daughter cells. The number of cell-lineages are drawn on each blastomere. Scale bar: 50 μm.

**FIGURE 4:**
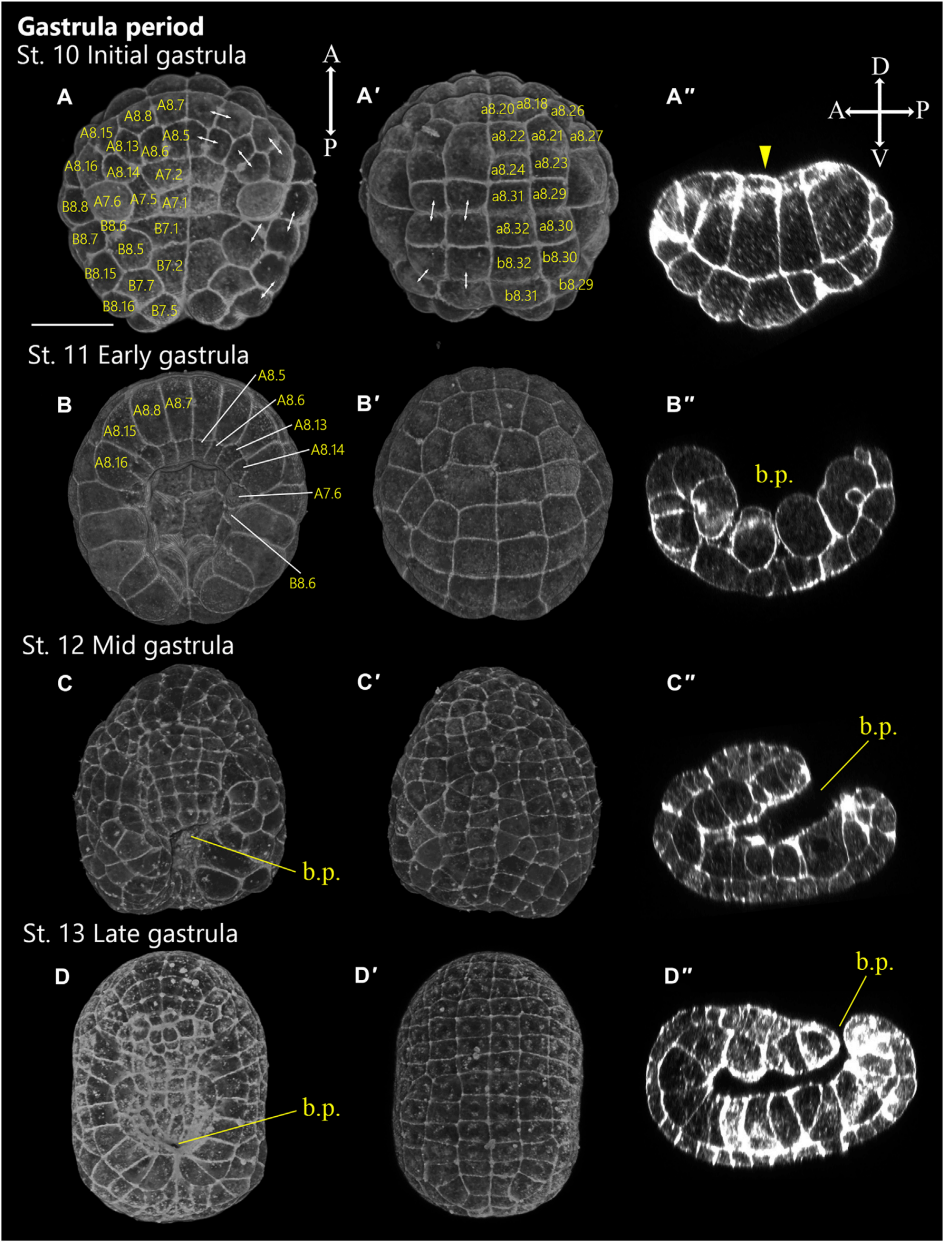
*Ascidiella aspersa* embryos at gastrula period. Embryos are stained with Alexa Fluor™ 546 Phalloidin. Three-dimensional reconstructed images at **(A,A′)** Stage 10 (110 cell, initial gastrula), **(B,B′)** Stage 11 (early gastrula), **(C,C′)** Stage 12 (mid gastrula), **(D,D′)** Stage 13 (late gastrula). **(A–D)** show the dorsal views of the embryos, and **(A′–D′)** show the ventral views of the embryos. **(A′′–D′′)** Longitudinal sections of **(A–D)**, respectively. Anterior side of each embryo is up in **(A–D, A′–D′)**. In **(A′′–D′′)**, embryo direction is shown by A, anterior, P, posterior, D, dorsal, V, ventral. Two-headed arrows indicate the pair of daughter cells. The number of cell-lineages are drawn on each blastomere. b.p., blastopore. Scale bar: 50 μm.

**FIGURE 5:**
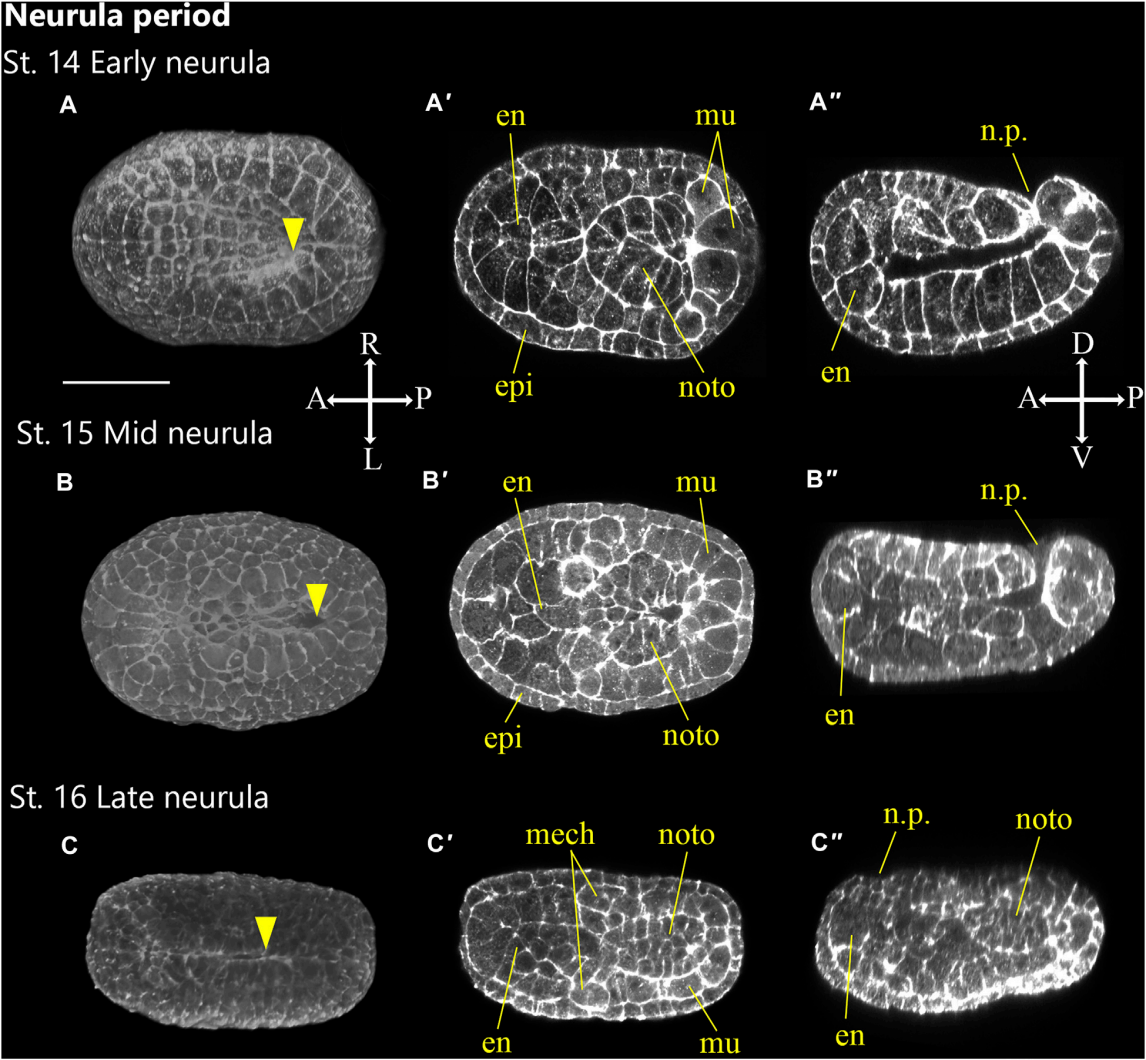
*Ascidiella aspersa* embryos at neurula period. Embryos are stained with Alexa Fluor™ 546 Phalloidin. Three-dimensional reconstructed images at **(A)** Stage 14 (early neurula), **(B)** Stage 15 (mid neurula), and **(C)** Stage 16 (late neurula). Three-dimensional reconstructed images at **(A)** Stage 14 (early neurula), **(B)** Stage 15 (mid neurula), and **(C)** Stage 16 (late neurula). **(A–C)** show the dorsal views of the embryos. **(A′–C′)** show the cross-section, and **(A′′–C′′)** show longitudinal section of **(A–C)**, respectively. Embryo direction is shown by A, anterior; P, posterior; D, dorsal, and V, ventral. Arrowheads indicates the anterior edge of neural tube closure. en, endoderm; epi, epidermis; mech, mesenchyme; mu, muscle; noto, notochord; n.p., neuropore. Scale bar: 50 μm.

**FIGURE 6:**
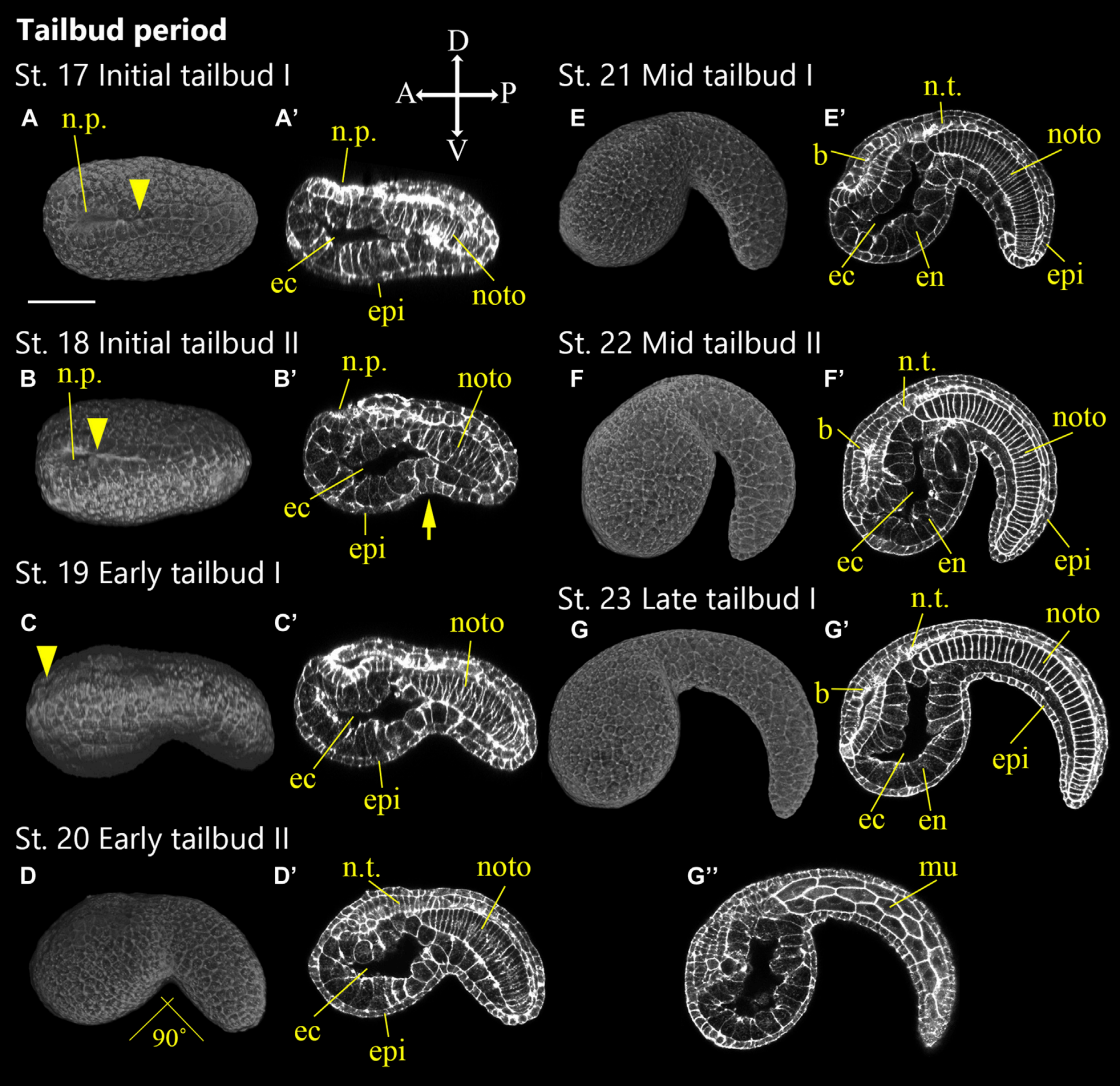
*Ascidiella aspersa* embryos at the first part of tailbud period. Embryos are stained with Alexa Fluor™ 546 Phalloidin. Three-dimensional reconstructed images at **(A)** Stage 17 (initial tailbud I), **(B)** Stage 18 (initial tailbud II), **(C)** Stage 19 (early tailbud I), **(D)** Stage 20 (early tailbud II), **(E)** Stage 21 (mid tailbud I), **(F)** Stage 22 (mid tailbud II), **(G)** Stage 23 (late tailbud I). **(A,B)** show the dorsal views of the embryos, and **(C–G)** show the lateral views of the embryos. **(A′**–**G′,G′′)** Longitudinal section of **(A–G)**, respectively. **(G′′)** is more lateral section than **(G′)**. Anterior side of each embryo is left. In **(C–G, A′–G′,G′′)**, embryo direction is shown by A, anterior; P, posterior; D, dorsal; V, ventral. The arrow indicates separation between tail and trunk territories. Arrowheads indicates the anterior edge of neural tube closure. *b*, brain; ec, endodermal cavity; en, endoderm; epi, epidermis; mech, mesenchyme; mu, muscle; noto, notochord; n.p., neuropore; n.t., neural tube. Scale bar: 50 μm.

**FIGURE 7:**
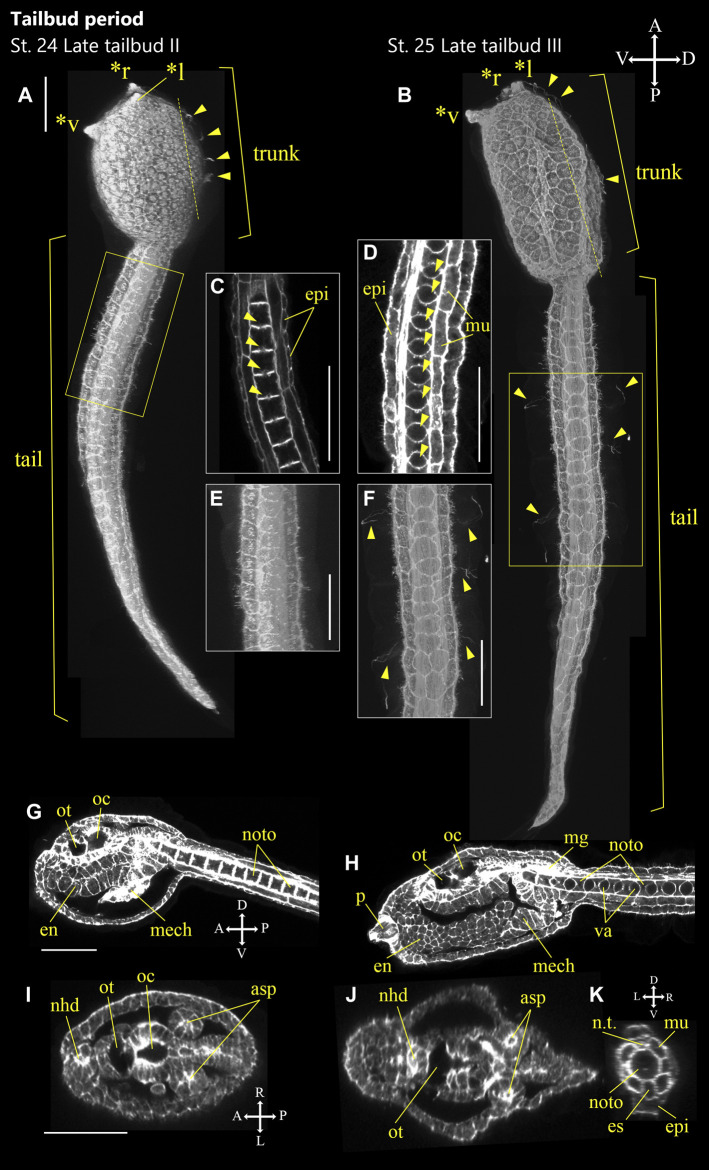
*Ascidiella aspersa* embryos at the latter of tailbud period. Embryos were stained with Alexa Fluor™ 54 Phalloidin. Three-dimensional reconstructed images at **(A)** Stage 24 (late tailbud II) and **(B)** Stage 25 (late tailbud III). **(A,B)** show the lateral views of the embryos. **(C,D)** Enlarged longitudinal section of the area inside the yellow frame in **(A,B)**, respectively. Arrowheads indicate vacuoles in notochord cells. **(E,F)** Enlarged image of the area inside the yellow frame in **(A,B)**, respectively. **(G,H)** Longitudinal section of **(A,B)**, respectively. **(I,J)** Cross-section of the trunk. The positions of the sections are indicated by dashed lines in **(A,B)**. **(K)** Cross-section of the tail in **(B)**. Embryo direction is shown by A, anterior; P, posterior; D, dorsal; V, ventral. In **(A,B, F)**, arrowheads indicate the cilia of epidermal sensory neurons. Asterisks indicates the papillae by *l: left papilla, *r: right papilla, *v: ventral papilla. asp, atrial siphon primordium; en, endoderm; epi, epidermis; es, endodermal strand; mech: mesenchyme; mg, motor ganglion; mu, muscle; nhd, neurohypophyseal duct; noto, notochord; n.t., neural tube; oc, ocellus; ot, otolith; p, papilla; and va, vacuole. Scale bar: 50 μm.

**FIGURE 8:**
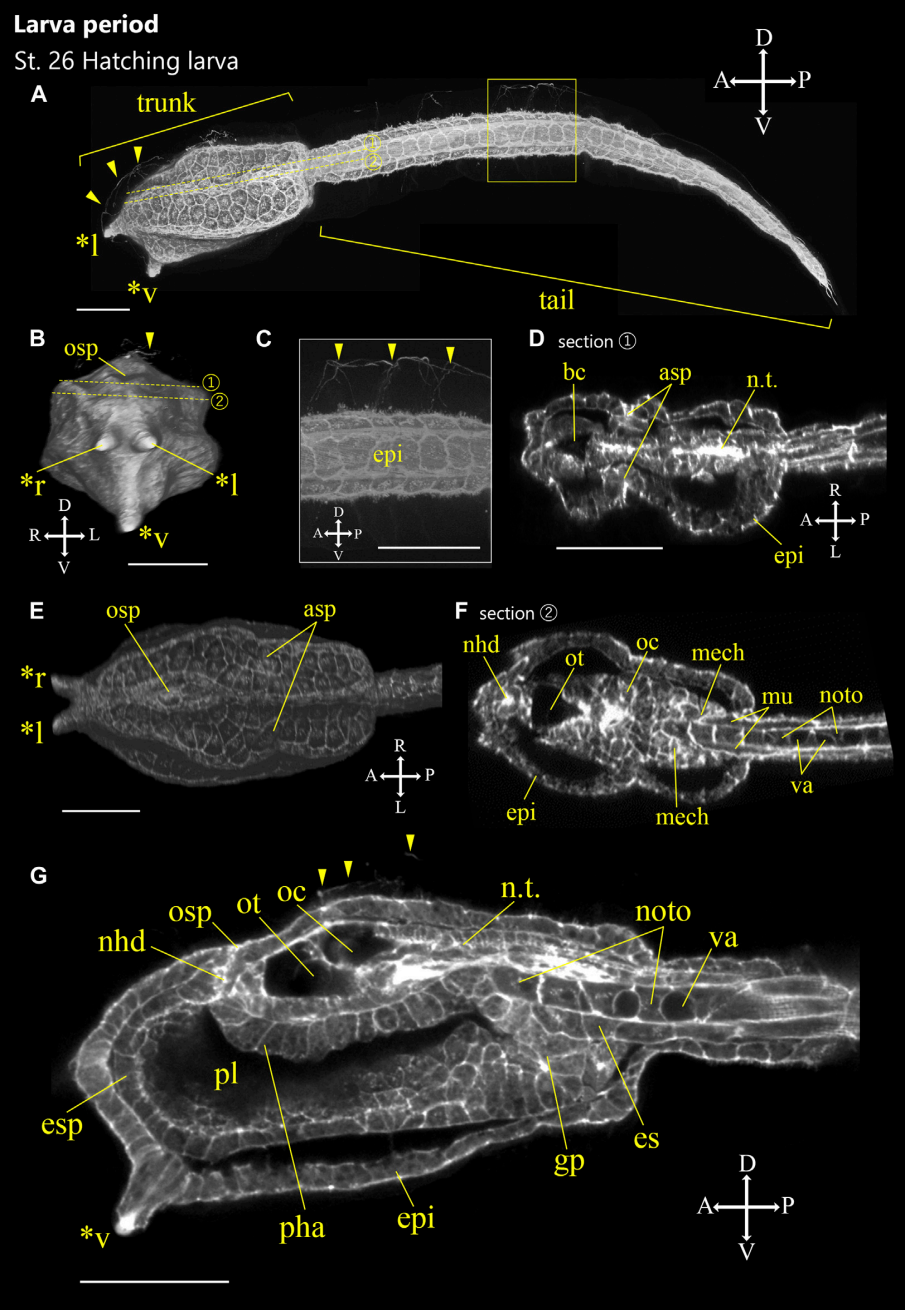
*Ascidiella aspersa* embryos at the initial part of larva period. Embryos were stained with Alexa Fluor™ 546 Phalloidin. Three-dimensional reconstructed images at **(A)** Stage 26 (hatching larva). **(A)** Lateral view of the embryo. **(B)** Frontal view of the embryo. **(C)** Enlarged image of the area inside the yellow frame in **(A)**. **(D,F)** Cross-section of the trunk. The positions of the sections are indicated by dashed lines in **(A,B)**. **(E)** Dorsal view of the trunk. **(G)** Longitudinal section of the trunk. Embryo direction is shown by A, anterior; P, posterior; D, dorsal; V, ventral. Arrowheads indicate the cilia of epidermal sensory neurons. Asterisks indicates the papillae by *l: left papilla, *r: right papilla, *v: ventral papilla. asp, atrial siphon primordium; bc, brain cavity; en, endoderm; epi, epidermis; es, endodermal strand; gp, gut primordium; mech: mesenchyme; mu, muscle; nhd, neurohypophyseal duct; noto, notochord; n.t., neural tube; oc, ocellus; osp, oral siphon primordium; ot, otolith; *pha*, pharynx; pl, preoral lobe; and va, vacuole. Scale bar: 50 μm.

After fertilization, we redefined 26 stages for embryogenesis of *A*. *aspersa* (Supplementary Figure S1; Table 1) based on the staging definition used for *Ciona* (Hotta et al., 2007). The developmental stages describe the process from fertilization to hatching in cases without chorion (Figure 1). Table 1 shows the time required to reach each developmental stage at 20°C as well as tail and trunk length ratios (T/H ratio) and time ratio to hatch (% hatch). The hatching time (avg. 16 h and 6 min post fertilization) was examined by time-lapse imaging of the embryos with the chorion. Embryogenesis was separated into six periods (Table 1): periods of zygote (Figure 2), cleavage (Figures 2, 3), gastrula (Figure 4), neurula (Figure 5), tailbud (Figures 6, 7), and larva (Figure 8).

**TABLE 1:** Stages of Early Embryonic Development in *Ascidiella aspersa*. From the left column. A total of 26 stages were divided into six periods: “Characteristics” is mainly based on the observation under dissecting microscopy. “Measurement of embryos”: Time after fertilization (average at 20°C, in Stage 1, 4 to 24, *N* = 11; Stage 2 and 25, *N* = 9; Stage 3, *N* = 10; Stage 26, *N* = 2), % hatch = rate of T (min)/969 (min) and ratio of tail/trunk length.

Stage		Characteristics	Measurement of embryos		
			Time after fertilization	% Hatch	Tail/Head ratio
I. Zygote period					
St. 1	One cell	Zygote, fertilized egg	0	0	
II. Cleavage period					
St. 2	Two-cell	The embryo composed of twocells	1 h 12 min	7	
St. 3	Four-cell	The embryo composed of four cells	1 h 32 min	10	
St. 4	Eight-cell	The embryo composed of eight cells. The cell division plane separates the animal half from the vegetal half located at a slight oblique angle. Vegetal posterior cells are bigger than others	1 h 55 min	12	
St. 5a	Early 16-cell	The embryo composed of 16 cells. Blastomeres is uncompacted in this stage. B4.1 cells make the first unequal cleavage	2 h 19 min	14	
St. 5b	Late 16-cell	The embryo composed of 16 cells. Blastomeres have been compacted	2 h 31 min	16	
St. 6a	Early 32-cell	The embryo composed of 32 cells. Blastomeres are uncompacted in this stage. B5.2 cells make the second unequal cleavage	2 h 46 min	17	
St. 6b	Late 32-cell	The embryo composed of 32 cells. Blastomeres have been compacted	2 h 59 min	18	
St. 7	44-cell	The embryo is composed of 44 cells. The vegetal side blastomeres are bulging out. B6.3 cells make the third unequal cleavage.	3 h 17 min	20	
St. 8	64-cell	The embryo composed of 64 cells. Embryo has an almost circle shape from top view	3 h 35 min	22	
St. 9	76-cell	The embryo is composed of 76 cells. The embryo flattens on its vegetal side in preparation for gastrulation	3 h 57 min	24	
III. Gastrula period					
St. 10	112-cell, initial gastrula	Gastrulation starts with A7.1 blastomeres, which is the center of invagination. The vegetal cells are thicker and more columnar	4 h 22 min	27	
St. 11	Early gastrula	The notochord has invaginated. The vegetal side of the embryo has a horseshoe shape	4 h 46 min	30	
St. 12	Mid gastrula	The embryo has six-row neural plate. The blastopore is located posterior and still open	5 h 15 min	33	
St. 13	Late gastrula	The embryo elongates anteriorly. The blastopore is located posterior and nearly closed. The neural plate has more than six rows and a part of neural rows start to curve	5 h 41 min	35	
IV. Neurula period					
St. 14	Early neurula	Neural plate forms a furrow. The embryo has an oval shape. The furrow is not closed	6 h 13 min	38	
St. 15	Mid neurula	The neural tube has formed along most of its length. The embryo has an oval shape. The A-line neural plate also forms a neural fold	6 h 44 min	42	
St. 16	Late neurula	The neural tube closure starts in the posterior part. The embryo elongates more along A-P axis	7 h 21 min	45	
V. Tailbud period					
St. 17	Initial tailbud I	First indication of a separation between trunk and tail parts in this stage. The tail is not bent and boundary area between trunk and tail parts bulging out. The neural tube closure in the posterior territory finished and the neuropore move more anterior. None of the notochord cells finish intercalation	7 h 56 min	49	0.9
St. 18	Initial tailbud II	The tail is clearly distinguished from the trunk and begins to bend. The tail is shorter than the trunk. The neuropore is still opened	8 h 18 min	51	0.9
St. 19	Early tailbud I	The angle formed by trunk and tail is an acute angle and has the same length as the trunk. A few anterior notochord cells finish intercalation and the neuropore just close	8 h 46 min	54	1.0
St. 20	Early tailbud II	The tail bends around 80°–90° and a half of notochord cells finished intercalation. The neuropore has closed	9 h 14 min	57	1.2
St. 21	Mid tailbud I	The tail is 1.5-fold longer than the trunk and the angle formed by trunk and tail is an obtuse angle. Intercalation of the notochord cells is completed	9 h 41 min	60	1.5
St. 22	Mid tailbud II	The body curves circularly. The tail is most acutely bending. The otolith pigmentation has not yet occurred	10 h 7 min	63	1.8
St. 23	Late tailbud I	The pigmentation of the otolith starts. The relaxation of the tail starts as the tail elongates. The tail length is twice as long as trunk	10 h 32 min	65	2.0
St. 24	Late tailbud II	The notochord vacuolation begins partially and palps formation is initiated by the anterior trunk epidermis thickening and bulging. Tail straightens in its anterior part	12 h 8 min	75	2.8
St. 25	Late tailbud III	The ocellus melanization is observed. The cilia elongation from caudal epidermal neuron begins. All notochord cells have vacuoles	13 h 20 min	83	2.8
VI. Larva period					
St. 26	Hatching larva	Larvae are hatching. The trunk has an elongated rectangular shape	16 h 16 min	100	3.0

The criteria for staging up to Stage 10 depends on the embryo shape and the number of cells. The later criteria are based primarily on the shape of the embryo and the length of the tail and trunk (after Stage 15) as well as angular tail bend according to Hotta’s criteria (Hotta et al., 2007).

One of staging criteria was the “standard development time” (in hours)—the normalized time for development when incubated at 20°C. Previously reported optimal incubation temperatures range from 16°C to 20°C for *Ciona* staging (Hotta et al., 2007). The hatching time after fertilization is slightly later than *Ciona* (16.1 hpf and 15.6 hpf at 20°C in *A*. *aspersa* and *Ciona*, respectively; Supplementary Figure S2).

### Zygote Period (0–1.2 h Post-fertilization at 20°C, Stage 1)

The zygote period (0–1.2 h post-fertilization at 20°C) was composed of one stage: Stage 1 extends from fertilization to the end of the first mitotic cell division (Figure 2). Figures 2B,B′ (optical section) show the first cell division in progress. The boundary between the two daughter cells is constricted but not yet fully separated. The egg diameter is relatively larger (175.4 ± 3.3 μm, *N* = 10) than that of *Ciona* (145.1 ± 1.8 μm, *N* = 10).

### Cleavage Period (1.2–4.4 h, Stages 2–9)

The cleavage period (1.2–4.4 h post-fertilization at 20°C) was composed of eight stages: Stages 2–9 (Figures 2C–3G″). The cleavage period is characterized by the formation of blastomeres through sequential mitosis. At these stages, the embryo is bilaterally symmetrical. In ascidians, unequal cell division (UCD) forms two small cells at the 16-cell stage followed by two successive UCDs. From the 16-cell stage, the mitotic cycle becomes asynchronous with the vegetative half dividing before the animal half producing the 24-, 32-, 44-, and 64-cell stages (McDougall et al., 2019). The criteria for staging during the cleavage period are not focused only on the number of cells but also on the embryo shape. During the early cleavage period (e.g., 16- and 32-cell stages) embryos change shape dynamically and quickly due to compaction. Thus, the 16-cell stage and 32-cell stage were divided into two sub-stages (16-cell stage: Stage 5a and Stage 5b; 32-cell stage: Stage 6a and Stage 6b, respectively).

#### Stage 2

Two-cell stage (1.2 h, Figures 2C,C′). The first cell division plane is located by dividing the embryo into left and right halves.

#### Stage 3

Four-cell stage (1.5 h; Figures 2D–E′). The second cell division plane divides the embryo into anterior and posterior halves. Immediately after cell division, each blastomere is loosely packed together (Figures 2D,D′), but the four blastomeres are compacted towards the end of the stage and the embryo appears circular (Figures 2E,E′). In *Ciona* Stage 3 (Four-cell stage), the centrosome-attracting body (CAB) structure (Hibino et al., 1998; Iseto and Nishida, 1999; Costache et al., 2017) could be easily recognized at the four-cell stage as the actin-thickening region of cortical membrane *via* phalloidin staining; this was difficult to recognize in *A*. *aspersa* (Figures 2D,D′).

#### Stage 4

Eight-cell stage (1.9 h; Figures 2F,F′). The third cell division plane separates the animal half from the vegetal half although it is located at a slight oblique angle not orthogonal to the first and second planes.

For the first time, four cell lineages are defined at this stage: a, animal anterior; b, animal posterior; A, vegetal anterior; B, vegetal posterior. It is possible to distinguish between the four cell lineages of blastomeres in living embryos by observing the size (Figures 2F,F′, B-line is biggest) and spatial placement of the cells. This characteristic asymmetric cell division is common to some ascidian species such as *C*. *intestinalis* and *Halocynthia roretzi* (Nishida, 1994; Ogura and Sasakura, 2013). The embryo compaction occurs towards the end of the stage, and the shape looks much like a sphere (see Supplementary Movie S1, *T* = 131 min).

#### Stage 5

16-cell stage (2.3 h; Figures 3A–B′′). All blastomeres undergo the fourth cell division synchronously. In *Ciona* and *Phallusia*, there is a clear anterior-posterior polarity in the shape of the embryo caused by the effect of CAB (Negishi et al., 2007; Dumollard et al., 2017): Although the effect of CAB on the anterior-posterior polarity is unknown in *A*. *aspersa*, the B5.2 blastomere is significantly smaller than the others (Figures 3A,B). This stage is separated into early, uncompacted, 16-cell stage (Stage 5a, Figures 3A–A′′) and a late, compacted, 16-cell stage (Stage 5b, Figures 3B–B′′).

#### Stage 6

32-cell stage (2.8 h, Figures 3C–D′′). All blastomeres undergo a fifth cell division, which occurs earlier in the vegetal than animal lineages.

The division of B5.2 cells is again remarkably asymmetric, thus producing small B6.3 cells at the posterior (Figures 3C′,C′′,D′,D′′). This asymmetric cell division is also influenced by CAB as the previous cell division in *Ciona* (Negishi et al., 2007). This stage is separated into an early 32-cell stage (Stage 6a; Figures 3C–C′′) in which the embryo is semi-spherical and a late 32-cell stage (Stage 6b; Figures 3D–D′′) in which the embryo is flat. The late 32-cell stage is characterized by a large expansion of B6.2 cells.

#### Stage 7

44-cell stage (3.3 h; Figures 3E,E′). All vegetal blastomeres have undergone their sixth cell division. The very small B7.6 blastomere is a marker of the posterior end of the embryo (Figure 3E).

#### Stage 8

64-cell stage (3.6 h; Figures 3F,F′). The animal side blastomeres also undergo the sixth cell division following the vegetal side. In the view from the animal pole, the embryo looks slightly angular than circular because the A7.8 and B7.4 blastomeres are protruding (Figure 3F; Supplementary Movie S1, *T* = 238 min).

#### Stage 9

76-cell stage (3.9 h; Figures 3G–G′′). Cell division is asymmetrical in the vegetal half of the embryo. The vegetal side of the embryo becomes flattened and is the prior phase of gastrulation (Figures 3G,G′′). The vegetal blastomeres are taller and more columnar than the animal cells (Figure 3G′′).

### Gastrula Period (4.4–6.2 h, Stages 10–13)

Gastrulation is a nearly universally conserved developmental process in animal embryogenesis during which dramatic morphological changes occur. During the seventh cell division, gastrulation begins when the large endoderm cells on the vegetal side of the embryo invaginate (Swalla, 1993). Ascidian gastrulation is initiated by the invagination of 10 endodermal precursor cells between the 64-cell stage and the late 112-cell stage. This process is driven in two steps by a myosin-dependent contraction of the actomyosin network in the absence of endodermal cell division (Sherrard et al., 2010; Winkley et al., 2019; Fiuza et al., 2020).

#### Stage 10

112-cell stage (4.4 h; Figures 4A–A′′); initial gastrula. Gastrulation starts with the apical constriction of A7.1 blastomeres (Figure 4A′′, arrowhead). The cells on the vegetal side are thicker and more columnar than those on the animal side (Figure 4A′′).

#### Stage 11

Early gastrula (4.8 h, Figures 4B–B′′); the notochord has invaginated. The endodermal cells become round, and the embryo looks like a horseshoe from the vegetal view (Figure 4B).

#### Stage 12

Mid gastrula (5.2 h, Figures 4C–C′′); six-row neural plate stage. Here, the blastopore is still open in the center of the embryo. The neural plate is flat and consists of six rows of cells (Three rows of A-line cells and three rows of A-line cells) arranged in a characteristic regular pattern.

#### Stage 13

Late gastrula (5.7 h, Figures 4D–D′′). The blastopore is located posterior of the embryo and is almost closed. The embryo develops along the anterior-posterior axis. The neural plate develops into six or more rows, and the A-line neural rows (I and II) start to curve (start of neurulation).

### Neurula Period (6.2–7.9 h, Stages 14–16)

Neurulation occurs during the neurula period. This is one of the defining events of chordate morphogenesis in which the neural tube forms and separates from a surface epidermis to form the rudiment of the future nervous system. The neurula period (Figures 5A–C′′) includes Stages 14–16.

#### Stage 14

Early neurula (6.2 h, Figures 5A–A′′): the neural plate forms a furrow (Figure 5A). The embryo of *A*. *aspersa* has a characteristic oval shape from the beginning of neurula period (Figure 5A; Supplementary Figure S2). The embryo of *Ciona*, on the other hand, has a diamond-like shape with the most acute anterior end. The neural fold is not yet closed (Figure 5A). Neural tube zippering starts from the posterior part of the embryo (Hashimoto et al., 2015).

#### Stage 15

Mid-neurula (6.7 h, Figures 5B–B′′). Most of the neural tube is still open. The shape of the embryo is oval, and the neural plate of the A-line cells also forms a neural fold (Figure 5B′′).

#### Stage 16

Late neurula (7.3 h, Figures 5C–C′′). The neural tube begins to close in the posterior region. The notochord precursor undergoes intercalation and convergence. Accordingly, the embryo becomes (Figures 5C–C′′) slightly longer than the Stage 15 embryo.

### Tailbud Period (7.9–16.1 h, Stages 17–25)

Stages 17–25 is defined as the tailbud period (Figures 6, 7; Table 1; Supplementary Figures S2) and is subdivided into four phases: initial tailbud, early tailbud, mid tailbud, and late tailbud stages. All tailbud stages, besides late tailbud, include two sub-stages representing the start and end of the stage. In every case, there is a clear visual marker at the start of the stage.

#### Stage 17

Initial tailbud I (7.9 h, Figures 6A,A′). Versus the *Ciona* initial tailbud I embryo, *A*. *aspersa* is characterized by the absence of a constriction where the tail and trunk regions are separated (Figure 6A). The tail is not obviously bent and is slightly shorter than the trunk (Table 1). In the posterior region, the neural tube closure is almost complete, and the neuropore moves toward a more anterior position (Figure 6A, n.p.). The notochord cells form two rows in the left-right side. Some of these are interdigitating but are still in intercalation (Figure 6A′, noto; see section mode of RAMNe).

#### Stage 18

Initial tailbud II (8.3 h, Figures 6B,B′). The tail begins to bend ventrally (Figure 6, arrow) so that the tail and trunk can be clearly distinguished. In the case of the same stage of *Ciona*, the tail and trunk have the same length, but the tail is shorter than the trunk in *A*. *aspersa* (Figures 6B,B′; Table 1). The neuropore can still be observed (Figures 6B,B′, n.p.).

#### Stage 19

Early tailbud I (8.8 h, Figures 6C,C′). The tail bends less than 90°, and the trunk separates from the tail and becomes spherical (Table 1) (in case of *Ciona* less than 60°). The intercalation of the few most anteriorly located notochord cells ends and the neuropore closes (Figure 6C′).

#### Stage 20

Early tailbud II (9.2 h, Figures 6D,D′). The tail bends about 80°–90°. The neuropore closes, and neurulation is complete at this stage (Figure 6D′).

#### Stage 21

Mid-tailbud I (9.7 h, Figures 6E,E′). The tail elongates 1.5-fold the length of the trunk (Table 1). The tail is curved more than 100°. The notochord cells finish intercalation (Figure 6E′, noto).

#### Stage 22

Mid-tailbud II (10.1 h, Figures 6F,F′). The entire body is semi-circular, and the tail bends most strongly at this stage. In *Ciona* Stage 22, the tail length is twice as long as trunk whereas *Aspersa* elongation is 1.8 times longer than the trunk (Table 1).

#### Stage 23

Late tailbud I (10.5 h, Figures 6G–G″). The onset of otolith pigmentation can be observed with a dissecting microscope (Supplementary Figure S2). In *Ciona*, the tail curvature is often stronger than the previous stage, but the curved tail starts to relax in accordance with the tail elongation in this stage in *A*. *aspersa*.

#### Stage 24

Late tailbud II (12.1 h, Figure 7). Vacuolation of notochord cells partially begins (Figure 7C, arrowheads). The palp develops by thickening and protrusion of epidermal cells in front of the trunk (Figure 7A, asterisks). The anterior part of the tail relaxes and straightens.

#### Stage 25

Late tailbud III (13.3 h, Figure 7). Melanization of ocellus is visible (Supplementary Figure S2). Cilia from the caudal epidermal neuron start to develop. (Figures 7B,F arrowheads). Three palps (left, right, and ventral) are recognized (Figure 7B asterisks). Vacuolization occurs in all notochord cells (Figure 7D). The tail relaxes and straightens.

### Larva Period (16.1 h)

#### Stage 26

Hatching larva (16.1 h, Figure 8). The trunk has an elongated rectangular shape (Figure 8A; Supplemenatry Figure S2). Three tips of the palps elongate (Figure 8, asterisks), and notochord vacuoles become larger (Figure 8). Cilia are grown from trunk epidermal sensory neuron, the apical trunk epidermal neuron (ATEN; Imai and Meinertzhagen, 2007), and the palp neuron in the larval trunk (Figure 8A, arrowheads). Cilia of the caudal epidermal sensory neuron are also elongated (Figure 8C, arrowheads). These cilia project into the fin tunic (Pasini et al., 2006; Terakubo et al., 2010; Yokoyama et al., 2014). A pair of atrial siphon primordia are obvious (Figure 8D, asp). Notochord cells have larger vacuoles. Otolith and ocellus melanization are well observed (Figure 8G; Supplementary Figure S1). Neurohypophyseal ducts can be recognized with oral siphon primordium but are not yet open (Figures 8F,G, nhd).

### Resources of *Ascidiella aspersa* Morphology for Network-Based

**FIGURE 9:**
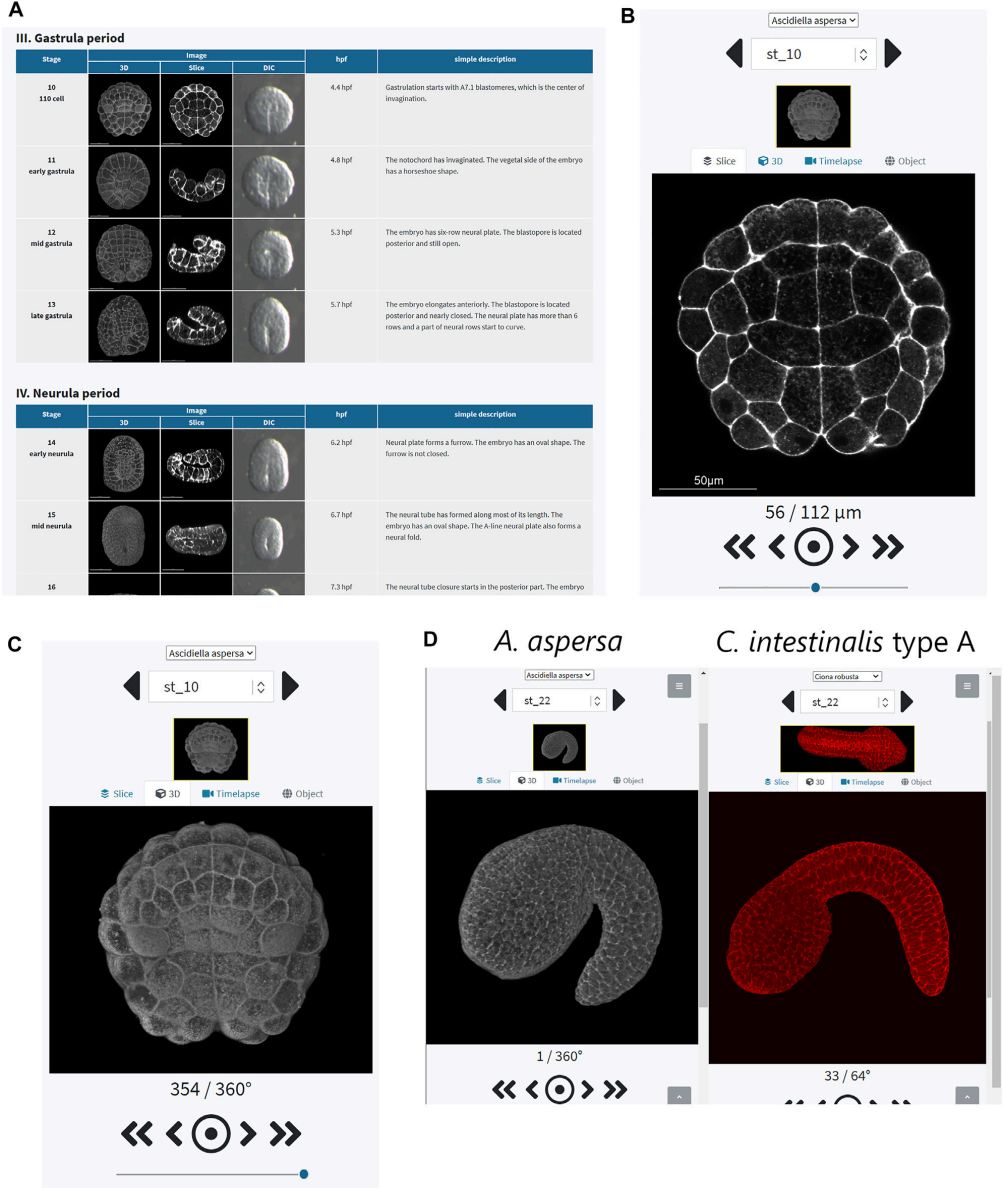
Screenshot of the database. To use the database, please refer to the website: https://www.bpni.bio.keio.ac.jp/RAMNe/latest/index.html. **(A)** “Developmental table” refers to the two-dimensional images from a fertilized egg to the hatched larva as viewed by CLSM and Nomarski. **(B)** Information about section images. **(C)** Information about 3D images. **(D)** “Dual display mode” can display and compare the images of *A*. *aspersa* and compared to *C*. *intestinalis* type A (Hotta et al., 2007; Hotta et al., 2020) interactively.

Images of *A*. *aspersa* embryos were exported as a series of image files so that both cross-sectional and 3D images could be easily viewed. These were visible *via* a web-browser. Images were linked to information on developmental stage, developmental nomenclature, hour post-fertilization (hpf), % hatch, cell lineage, and time-lapse movies. We integrated these data into a web-based database, “R*A*MNe.” The user can observe the 3D images interactively and for each z-section image online (Figure 9; https://www.bpni.bio.keio.ac.jp/RAMNe/latest/index.html). When selecting any developmental stage from the developmental table (Figure 9A), users can easily view 3D- (Figure 9C) and Z-section (Figure 9B) images of the entire embryo at that stage. In the “z-section module,” users can display all the section images between the selected focus ranges using a slider (Figure 9B). The “rotation module” allows one to view the 3D image of the embryo rotated along the *Y*-axis in 1° angle steps. One of the unique features of the R*A*MNe is that corresponding images from *Ciona* can also be displayed (Hotta et al., 2007, Hotta et al., 2020). This can then be interactively compared with *A*. *aspersa* (Figure 9D). In the download section, users can freely download resource files under the terms of the Creative Commons Attribution License (CC BY). All pages are adaptive for viewing on various devices and are free to access anytime; anyone can freely access the ascidian morphology resource.

## Discussion

We defined staging of *A*. *aspersa* (early embryogenesis to hatching larva) *via* CLSM and time-lapse imaging. According to previously defined developmental stages in *C*. *intestinalis* type A (Hotta et al., 2007, Hotta et al., 2020), we defined the standard developmental staging of *A*. *aspersa*. The criteria were redefined by descriptions of *A*. *aspersa* morphology, time after fertilization at 20°C, and ratio until hatching (Table 1). We prepared images of both by stereomicroscope (Supplementary Figure S1) and by CLSM. These relate to each other in each stage and explain the inner structure of the embryo at each stage.

### Difference of Ascidiella *aspersa* Morphogenesis With *Ciona*

**FIGURE 10:**
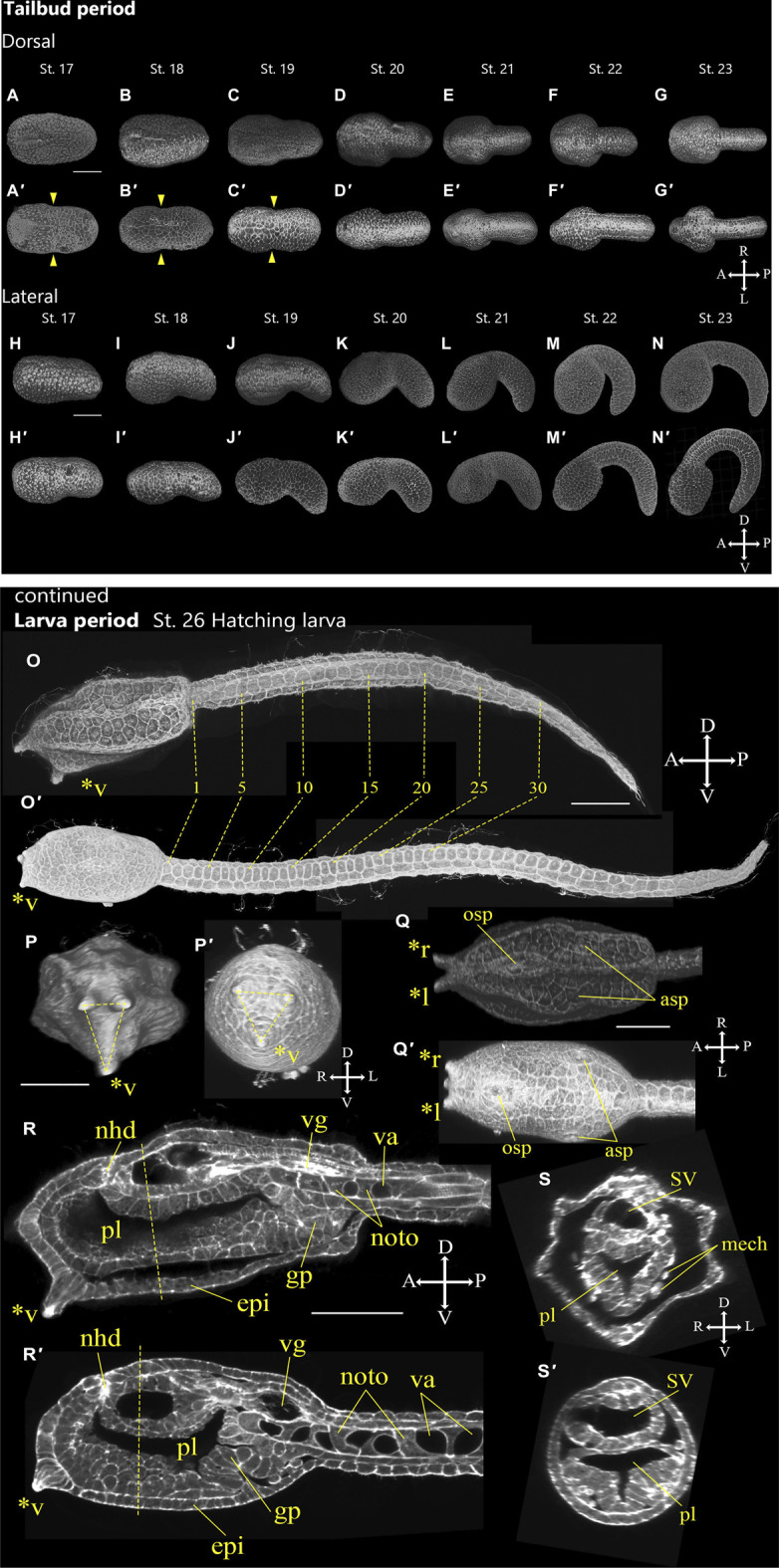
Comparison of tail bending and KUBIRE at tailbud period and larval morphology between *A*. *aspersa* and *C*. *intestinalis*. Dorsal views of the *A*. *aspersa* embryos **(A–G)** and the *C*. *intestinalis* embryos **(A′–G′)** at Stage 17–23. Lateral views of the *A*. *aspersa* embryos **(H–N)** and the *C*. *intestinalis* embryos **(H′–N′)** at Stage 17–23. Larval morphology of *A*. *aspersa* **(O–S)** and *C*. *intestinalis* **(O′**–**S′)** at Stage 26. Embryo direction is shown by A, anterior; P, posterior; D, dorsal; V, ventral. Arrowheads indicate hour-grass shaped KUBIRE. Asterisks indicates the papillae by *l: left papilla, *r: right papilla, *v: ventral papilla. asp, atrial siphon primordium; epi, epidermis; gp, gut primordium; mech, mesenchyme; nhd, neurohypophyseal duct; noto, notochord; osp, oral siphon primordium; pl, preoral lobe; SV, sensory vesicle; va, vacuole; and vg, visceral ganglion. Scale bar: 50 μm.

**FIGURE 11:**
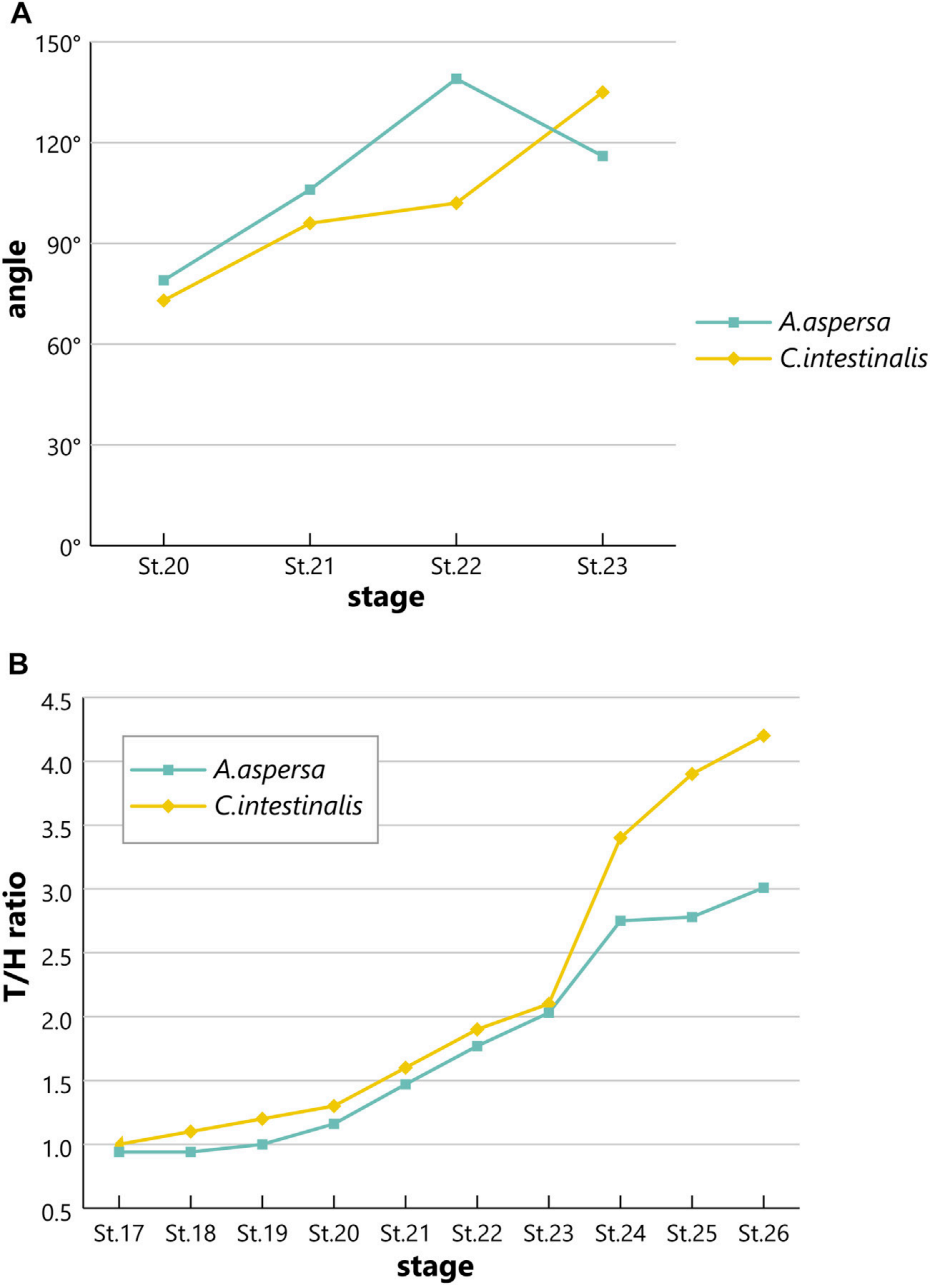
Comparison of bending angle or tail/head ratio between *A*. *aspersa* and *C*. *intestinalis* type A at tailbud and larval periods. **(A)** Bending angle between the tail and the trunk at Stage 20–23. **(B)** The ratio of the trunk and tail length at Stages 17–26.

Although *Ciona* and *A*. *aspersa* shared stereotyped development with conserved cleavage patterns and developmental timing, our detailed observation shows few differences between them. *A*. *aspersa* has a relatively shorter tail and larger trunk with *Ciona*. This indicates different body shapes in some stages. For example, the embryo was ovular in *A*. *aspersa* at Stage 14 (Figure 5A; Supplementary Figure S1) but diamond-shaped in *Ciona*. The initial tailbud embryos from Stage 17—*Ciona* have an hourglass-like epithelial bending between trunk and tail regions. This is similar to “KUBIRE” in Japanese (Nakamoto and Kumano, 2020) whereas no KUBIRE is seen in *A*. *aspersa* (7.9 h, Figures 6A,A′, 10). This KUBIRE shape was made by different orientation of cell division in the transition between trunk and tail. To understand how such different body shapes are created, it will be interesting to investigate the orientation of cell division responsible for making KUBIRE in *A*. *aspersa*. However, the ratio of the trunk and the tail length in the tailbud period was smaller than that of *Ciona* indicating a relatively short tail (Figure 11B). At Stage 22 and Stage 26, the tail length is 1.9 and 4.2 times of the trunk, respectively (Hotta et al., 2007). These values are 1.8 and 3.0 times, respectively, in *A*. *aspersa*.

The two species in the tailbud stages show tail curving in the tailbud embryo, but the timing of the relaxation of the tail bending is relatively earlier than that of *Ciona* (Figure 11A), and the maximum curvature of *A*. *aspersa* in tailbud stages is weaker than that of *Ciona* (Figure 6; see R*A*MNe using dual view). Tailbud embryo tail bending is controlled by asymmetric actomyosin localization in notochord at early tailbud stages (Lu et al., 2020); *Admp* regulates the tail bending (Kogure et al., 2021, submitted). The differences in the tail bending curvature among the two species might be regulated by the different spatio-temporal expression of these molecules.

Some morphological differences are also observed between the two species in the larva period. The ventral papilla in *A*. *aspersa* is located more ventral than *Ciona* (Figures 10O,O′). Three papillae in *Ciona* form an equilateral triangle from the frontal view. On the other hand, *A*. *aspersa* forms an isosceles triangle (Figures 10P,P′). *A*. *aspersa* larva have around 30 lateral epidermal cells [fewer than *Ciona* (Figures 10O,O′)]. This lower number of tail epidermis cells might reduce the surface area of the tail and lead to a shorter tail in *A*. *aspersa*. In *A*.*aspersa*, two atrial siphon primordia are located in the dorsal trunk, but they are located in the lateral trunk in *Ciona* (Figures 10Q,Q′).

The short tail in *A*.*aspersa* compared to *Ciona* may reflect divergence of ascidian larva morphology and ecology. Short tails related to the reduction of the surface area of the tail can lead to a decline in the tadpole swimming (Van Buskirk and Mccollum, 2000). Upward and downward swimming with optical and gravity sensors can offer important insight into habitat and selection of substratum (Kajiwara and Yoshida, 1985; Bostwick et al., 2020): Weak swimming predicts poor dispersion despite the considerable mobility and invasiveness of this species (Lynch et al., 2016). However, short tail ascidian may have advantages in terms of metamorphosis speed as in no-tail ascidian, *Molgula* (Fodor et al., 2021). Investigations of the divergence in larval morphology can encourage a better understand of ecology-evolution-development studies in ascidians.

### Robust Mechanism of Developmental Speed

The egg diameter of *A*. *aspersa* is relatively larger (175.4 ± 3.3 μm, *N* = 10) than that of *Ciona* (145.1 ± 1.8 μm, *N* = 10). The timing of cleavage (∼Stage 12) and subsequent morphogenesis, gastrulation, neurulation, tail elongation, and the hatching time of *A*. *aspersa* after fertilization were nearly the same as *Ciona* (Supplementary Figure S2) if incubated at the same temperature. The growth curves of the two species are also very similar at the same temperature (20°C; Supplementary Figure S2) confirming that the developmental stages of both species correspond to each other. The ratio of each period was also conserved among both species. A previous dwarf embryo experiment was produced by artificial egg size reduction (Matsumura et al., 2020) and indicated that ascidian developmental speed was robust regardless of embryonic size. At least two Ascidian species have an evolutionarily robust mechanism for development speed regardless of the embryo size.

### Widely Used Model Organisms

*A*. *aspersa* has a big advantage in bioimaging research because of its extremely transparent embryo (Shito et al., 2020). This feature is unique to Ascidians of the family Ascidiidae such as *Phallsuia* and *A*. *aspersa* embryos, which are not found in *Ciona* and can help visualize embryos in three dimensions (Supplementary Movie S2). Moreover, remarkable feature of *A*. *aspersa* is the ability to do quick translation of external mRNA in the unfertilized egg (Yasuo and McDougall, 2018). To demonstrate this, we show the Ca^2+^ elevation at the fertilization by using genetic Ca^2+^ sensor, GCaMP6s (Supplementary Movie S3). As previously reported in *Ciona savignyi* and *A*. *aspersa* by using chemical Ca^2+^ sensor (Yoshida et al., 1998; Levasseur and McDougall, 2000), two series of Ca^2+^ oscillations were also observed in *A*. *aspersa* (Supplementary Figure S3). This demonstrates usefulness as a model animal for bioimaging and the potential benefits of using a variety of genetic sensors to visualize biological events early in their development.

However, there are still several points to note. Unlike *Ciona*, eggs are considerably more sensitive when dechorionated; fertilization is more difficult, and they are considerably more seasonal. The reason for the sensitivity in fertilization may be contamination of highly acidic body fluids. Isolation of gametes should involve gonoduct flushing with ASW to remove acidic fluids as shown by Bolton and Havenhand (Bolton and Havenhand, 1996). *A*. *aspersa* is continuous breeder year-round, but individuals with mature gonads are most abundant in the winter and spring and lowest in the summer (Lynch et al., 2016). Improved methods of handling and egg availability will promote bioimaging research with *A*. *aspersa*.

### System Level Understanding of Morphology and Evolution in Invertebrates Chordates Ascidians

Our precise description of anatomy and developmental staging for *A*. *aspersa* in this study, along with the ontology for anatomy and development, leads to a standard developmental table of *A*. *aspersa* for the scientific community including evo-devo, developmental biology, ecology, and cell biology. The embryos imaged in this study did not have chorion for the clear observation but the dechorionation was known to cause a mild effect on the morphogenesis (Oonuma et al., 2016; Kourakis et al., 2021). Thus, we expect chorionated specimens of *A*. *aspersa* development in subsequent versions of our R*A*MNe database.

Our embryo-imaging resource has value in creating a 3D standard model based on the real stack images for quantitative approach (Robin et al., 2011a; Nakamura et al., 2012; Veeman and Reeves, 2015; Matsumura et al., 2020). *Ciona* and *Phallusia* have good developmental 3D image resources that are very useful to understand morphometrical information of each embryo/blastomere (e.g., FABA (https://www.bpni.bio.keio.ac.jp/chordate/faba/1.4/top.html), TunicAnatO (https://www.bpni.bio.keio.ac.jp/tunicanato/3.0/index.html), and ANISEED database (https://www.aniseed.cnrs.fr/) (Hotta et al., 2007; Brozovic et al., 2016; Brozovic et al., 2018; Hotta et al., 2020).

The morphology in the stages before tailbud of *A*. *aspersa* is similar to that one of *Ciona*. Guignard suggested considerable embryonic reproducibility with early embryonic cell lineages conserved between distantly related ascidian species may play a role in contact area-dependent cell inductions during animal embryogenesis instead of morphogen gradients. These may relax constraints on genome evolution in ascidians (Guignard et al., 2020). Resource data of multiple ascidian embryo morphology can verify these hypotheses and provide valuable insight into the mechanism of deep conservation of morphology as well as the evolution of system-level morphogenesis in embryonic development.

We hope that the developmental staging and anatomy of *A*. *aspersa via* R*A*MNe will facilitate the use of this fascinating animal as a model animal for SLUMEICA.

## Experimental Procedures

### Biological Materials and Preparation of Embryos

*A*. *aspersa* adults were obtained from Onagawa Field Center or Hakodate Fisheries Research Institute. Eggs and sperm were isolated from the gonoducts. Chorion surrounding eggs were removed enzymatically according to a previous protocol (Hotta et al., 1999). Dechorionation of eggs was performed with the solution 0.05% actinase-E and 1% mercaptoacetic acid sodium salt in ASW as in *Ciona* (Hotta et al., 1999). The time of the dechorionation was relatively longer and took 10–20 min. Eggs and embryos were incubated with Millipore-filtered seawater (MFSW) on the 0.1% gelatin-coated dishes. Embryos were incubated at 20°C after fertilization.

### Time-Lapse Imaging by Stereomicroscopy

A Peltier-based thermo-stage (TOKAI-Hit) was placed on the microscope stage (OLYMPUS SZX16); the temperature was stabilized and maintained. The embryo was placed on a thermal plate and maintained during observation to stabilize the temperature at 20°C to acquire images. Images were acquired every one or 3 min.

### Fixed Embryo Image Collection by Confocal Laser Scanning Microscopy

We fixed embryos every 10–30 min from fertilized eggs to hatching larva stage. The method of the fixation was described previously (Hotta et al., 2007). Distinct representative embryos in each stage were chosen based on the *Ciona* staging criteria (Hotta et al., 2007) from CLSM data (Figure 1). We used an 40× oil-immersion objective lens (NA: 0.75) and set the confocal aperture to 140 μm. The size of the images was set to 1,024 px × 1,024 px. The size of the pixels was 0.129–0.31 μm/pixel in *X* and *Y* directions. The scanning interval in the *z*-axis direction was set to 1 μm. Cortical actin filaments was stained by using Alexa 546 phalloidin. All 3D images were reconstructed from around 100 sectioned images.

### Live Imaging of the Ca^2+^ Oscillation at Fertilization

The same method of visualization of Ca^2+^ for *Ciona* embryo was used (Akahoshi et al., 2017). In brief, the pSPE3- GCaMP6s plasmid was linearized using SfiIor DraIII, and GCaMP6s mRNA was produced and precipitated using the mMESSAGE mMACHINE T3 kit (Life Technologies, Carlsbad, CA, United States) following the manufacturer’s protocol. GCaMP6s mRNA was injected into dechorionated *A*. *aspersa* eggs at 0.5 μg/μl. The mRNA-injected egg was incubated in a 3-cm glass base dish at 20°C for more than 3 hours. Spontaneous fertilization was observed under fluorescence microscopy after adding one drop of sperm.

### Live Imaging of the Plasma Membrane

FM4-64 (Invitrogen) was used to visualize plasma membranes. Embryos were incubated in seawater with the final concentration of FM4-64 10 μM. Under CLSM, Laser excitation at 559 nm was used to visualize the signals in FM4-64. The size of the images was set to 512 px × 512 px. The size of the pixels was 0.564 μm/pixel in *X* and *Y* directions. The scanning interval in the *z*-axis direction was set to 4 μm. The z-stack consists of 36 images was taken every 2 min.

## Data Availability

The datasets presented in this study can be found in online repositories. The names of the repository/repositories and accession number(s) can be found in the article/Supplementary Material.

## Supplementary Material

Supplementary Figure 1 Early embryonic development of *A.* aspersa. Scale bar: 50 μm.

Supplementary Figure 2 Relationships between the time after fertilization and developmental stage in *A. aspersa* (20°C) and *C. intestinalis* type A. Developmental time course plot of *Ascidiella aspersa* embryos and larvae at 20°C.

Supplementary Figure 3 Ca^2+^ dynamics of an inseminated egg of *A. aspersa*. Images were collected every 1 s using fluorescent microscopy and displayed every 3 s in this figure. Two series of Ca^2+^ transients were observed immediately after fertilization. **(A)** First phase of Ca^2+^ dynamics. The Ca^2+^ increase propagated from the place indicated by an arrowhead. pb, polar body. **(B)** Second phase of Ca^2+^ dynamics. **(C)** The change of relative fluorescence intensity of GCaMP6s during Phase I and Phase II of fertilization.

Supplementary Movie 1 Time-lapse movie of *A. aspersa* development at 20°C.

Supplementary Movie 2 Three-dimensional timelapse imaging of *A. aspersa* embryo development. The plasma membrane was stained with FM4-64. Images are acquired by using 40× oil immersion lens, 559 nm excitation. Every z-stack image (consists of 22) with 3-µm intervals were taken every 3 s. Different embryo images from 32-cell stage to early tailbud stage were combined.

Supplementary Movie 3 Ca^2+^ imaging during fertilization of *A. aspersa*. Fluorescent GCaMP6s were acquired every 1 s using fluorescent microscopy.
