# Supplementary figures and images for: Developmental Table and Three-Dimensional Embryological Image Resource of the Ascidian Ascidiella aspersa
Source: Front Cell Dev Biol. 2021 Dec 17;9:789046. doi: 10.3389/fcell.2021.789046 (PMC8718802; doi:10.3389/fcell.2021.789046)

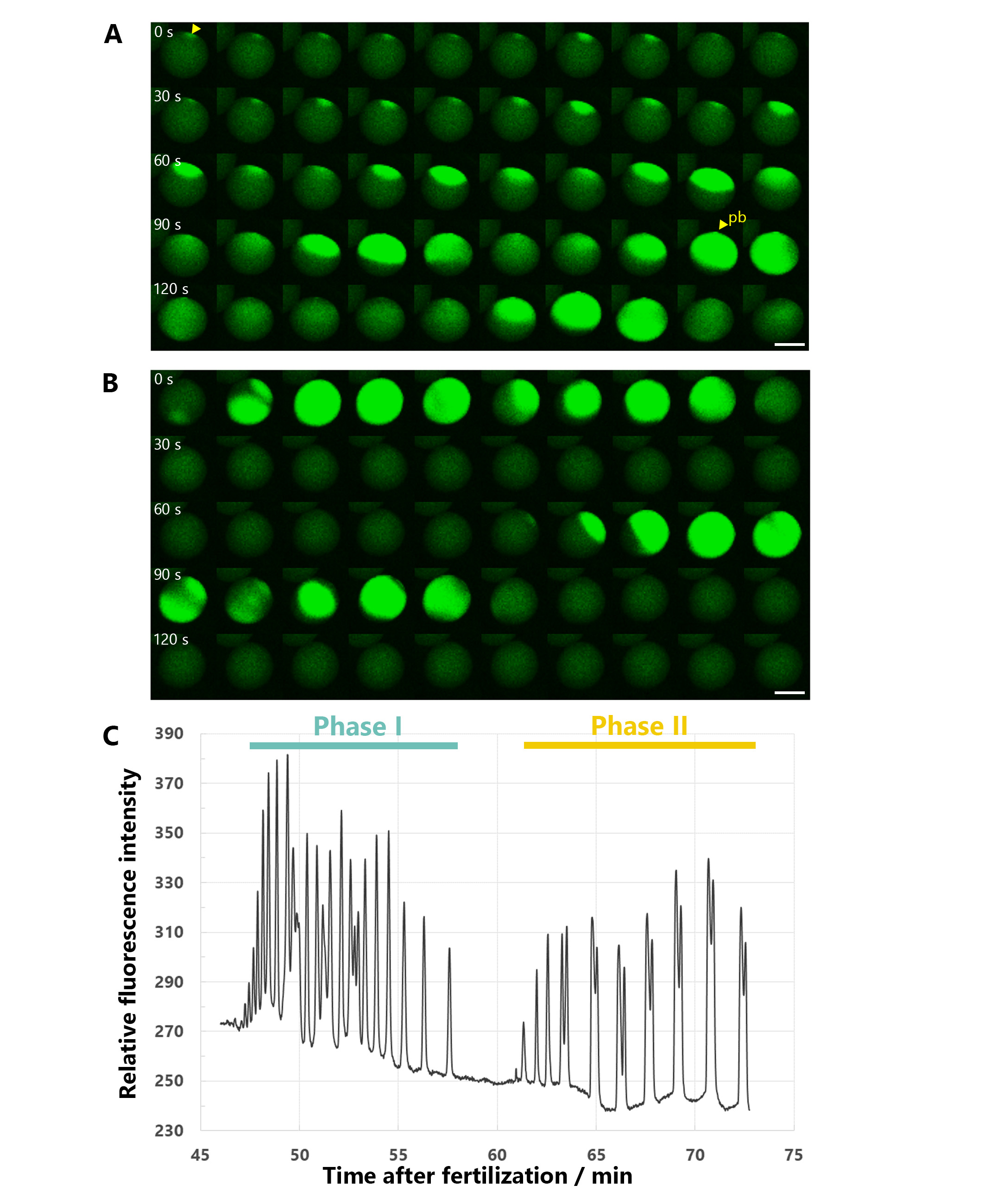

Supplement: Supplementary file 1 [file Image3.JPEG]

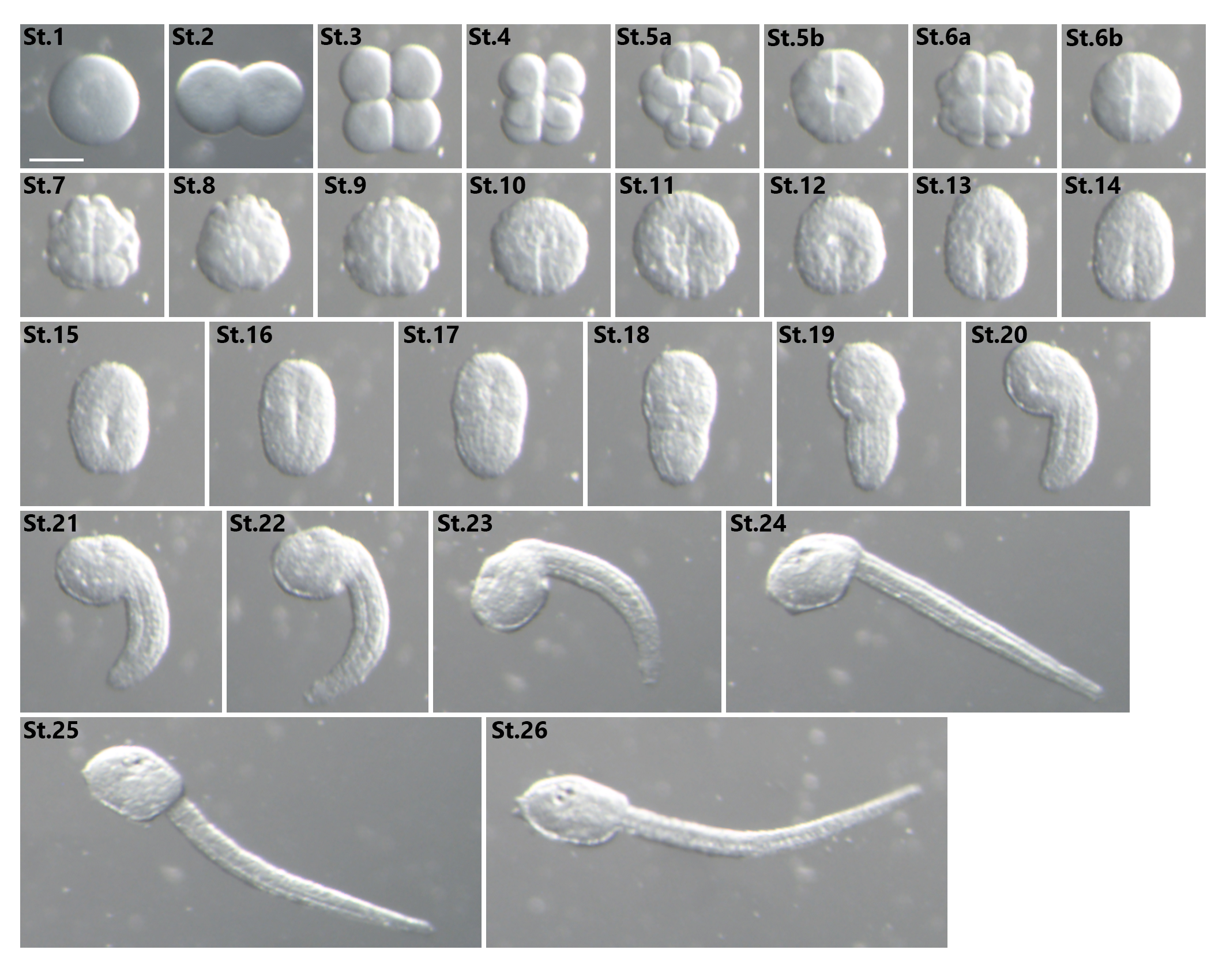

Supplement: Supplementary file 3 [file Image1.JPEG]

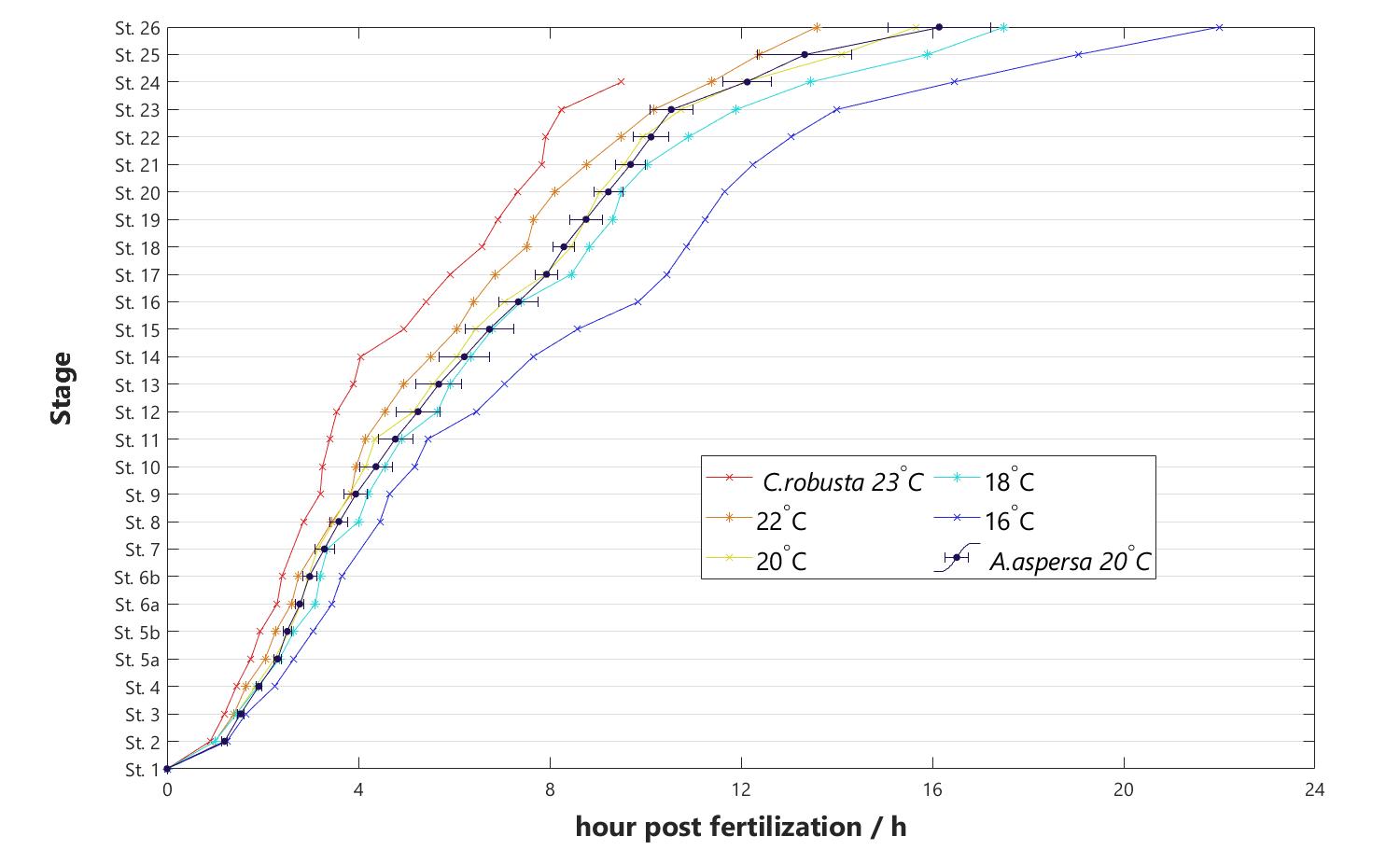

Supplement: Supplementary file 4 [file Image2.JPEG]
